# Serum Calprotectin is Associated with Overweight and Laboratory Markers of Glucose Metabolism in Apparently Healthy Young Adults—A Cross-Sectional Descriptive Study

**DOI:** 10.3390/metabo15120756

**Published:** 2025-11-21

**Authors:** Katarzyna Bergmann, Anna Stefańska, Magdalena Kuligowska-Prusińska, Magdalena Krintus

**Affiliations:** Department of Laboratory Medicine, Collegium Medicum in Bydgoszcz, Nicolaus Copernicus University in Toruń, 87-100 Toruń, Poland

**Keywords:** inflammation, insulin resistance, cardiometabolic risk

## Abstract

**Background**: Recent studies have indicated that serum calprotectin, a marker of inflammation, is associated with obesity and disorders of glucose and lipid metabolism. The aim of this study was to evaluate the relationship between serum calprotectin and cardiometabolic risk factors in presumably healthy young adults. **Methods**: The study enrolled 118 (61 females, 57 males) non-obese, normoglycemic, subjects aged 25–40 years, selected from the general population among participants of the diabetes preventive screening program in 2014–2015. Basic anthropometric measurements and the following laboratory tests were performed on all participants: glucose, glycated hemoglobin (HbA1c), lipid profile, insulin, Homeostatic Model Assessment for Insulin Resistance (HOMA-IR), high sensitivity C-reactive protein (hs-CRP), calprotectin and adiponectin. **Results**: The serum calprotectin concentration was significantly higher in men compared to women (*p* = 0.016), and in overweight subjects (*p* < 0.001) and those with abdominal obesity (*p* < 0.001), compared to lean individuals. Serum calprotectin was positively correlated with body mass index (BMI), waist circumference, HbA1c, hs-CRP, insulin, HOMA-IR and triglycerides, and negatively with HDL-cholesterol and adiponectin. In the univariable logistic regression analysis, overweight (OR = 2.529; *p* = 0.015), abdominal obesity (OR = 3.217; *p* = 0.006), hs-CRP > 1 mg/L (OR = 5.00; *p* < 0.001), HOMA-IR > 2.0 (OR = 4.394; *p* < 0.001), and HbA1c > 32 mmol/mol (OR = 2.166; *p* = 0.021) were significant predictors of increased calprotectin concentration (≥540.8 ng/mL; ≥median). However, in models adjusted for sex, BMI and hs-CRP, the significant association remained only for increased HbA1c and HOMA-IR values. **Conclusions**: Association of serum calprotectin with overweight, hs-CRP and laboratory indicators of glucose metabolism and insulin resistance suggest its significance as a laboratory biomarker of initial metabolic impairment.

## 1. Introduction

The increasing incidence of cardiometabolic disorders and the often-accompanying overweight and obesity in recent decades indicate the need for a thorough understanding of their pathological mechanisms and the search for new biomarkers that could be useful for the prevention and early diagnosis of metabolic disorders. One of the key mechanisms linking overweight and obesity with an increased risk of type 2 diabetes (T2DM) and cardiovascular disease (CVD) is low-grade inflammation, associated with the proinflammatory activity of adipose tissue [1]. The involvement of inflammatory factors in this process is very complex and multifaceted, including impairment of endothelial function, intensification of pro-oxidant reactions and overproduction of free radicals, and increased expression of other inflammatory and chemotactic molecules that enhance the influx of immune cells. The result of these processes is increasing insulin resistance, which impairs metabolic pathways and leads to hyperglycemia, dyslipidemia and hypertension [2,3]. For many years, research has emphasized the use of laboratory markers of inflammation to assess cardiometabolic risk, pointing to C-reactive protein (CRP), proinflammatory cytokines (e.g., interleukin 6 and 1; IL-6, IL-1) and adipose tissue-specific adipocytokines as potential diagnostic tools. While CRP and interleukin-6 have a well-established diagnostic significance, especially in the context of cardiovascular risk assessment [4], and their assay methods are widely available in medical laboratories, for other inflammatory markers there is still insufficient evidence to support their use in routine clinical practice, and assay methods are often difficult to access and not standardized.

Calprotectin (also called S100A8/A9, MRP8/14) is a heterodimeric protein binding Ca^2+^ and Zn^2+^ ions, released from neutrophils and monocytes during inflammation. Its role in inflammation involves stimulating chemotaxis and adhesion of inflammatory cells and inducing the production of reactive oxygen species. It may also exhibit antibacterial activity—by chelating metal ions, it prevents bacteria from utilizing them and inhibits their growth [5]. Although this protein has no organ specificity and its elevated concentration in blood should be considered as an indicator of systemic inflammation, in laboratory diagnostics the determination of fecal calprotectin is commonly used as a marker of inflammatory bowel disease (IBD). Numerous studies have shown that fecal calprotectin concentration strongly correlates with disease activity in Crohn’s disease and ulcerative colitis. The diagnostic performance, non-invasiveness, and rapidity of the test have allowed it to be used as an initial tool for suspected IBD and for differentiating from irritable bowel syndrome, significantly reducing the number of unnecessary endoscopies [6,7,8]. The diagnostic use of circulating calprotectin has been evaluated in studies of systemic connective tissue diseases. Increased serum calprotectin concentrations have been observed, among others, in the course of rheumatoid arthritis, systemic lupus erythematosus and juvenile idiopathic arthritis [9]. Alternatively, serum calprotectin can also be used as a marker of inflammation and a predictor of long-term mortality risk in critically ill patients with sepsis [10].

Due to the fact that calprotectin is secreted by neutrophils and macrophages, its presence in inflamed tissues may be a potential link with adipose tissue and metabolic disorders, and therefore, serum calprotectin levels may be a potential biomarker of cardiometabolic risk [11]. Although it has not been proven so far that calprotectin can be secreted directly by adipocytes, a study by Catalan et al. has shown increased expression of calprotectin in the stromavascular fraction cells of visceral adipose tissue (VAT) and its relationship with the content of monocytes and macrophages in VAT [12]. In an animal model study, higher mRNA expression of calprotectin subunits was also observed in mature adipocytes and in the stromal vascular cells of obese mice [13]. In humans, circulating calprotectin levels are significantly higher in obese individuals, correlate with body mass, fat content, CRP, indicators of insulin resistance [12] and are associated with diabetes, hypertension, poorer glycemic control, unfavorable lipid profile and increased risk of atherosclerotic cardiovascular disease [14]. However, there is still a lack of studies on large cohorts that would assess the usefulness of serum calprotectin as a biomarker of early metabolic disorders in young, clinically healthy individuals.

The aim of this preliminary study was to evaluate the relationship between serum calprotectin and selected cardiometabolic risk factors in apparently healthy, non-obese and normoglycemic young adults.

## 2. Materials and Methods

### 2.1. Study Group

**Figure 1:**
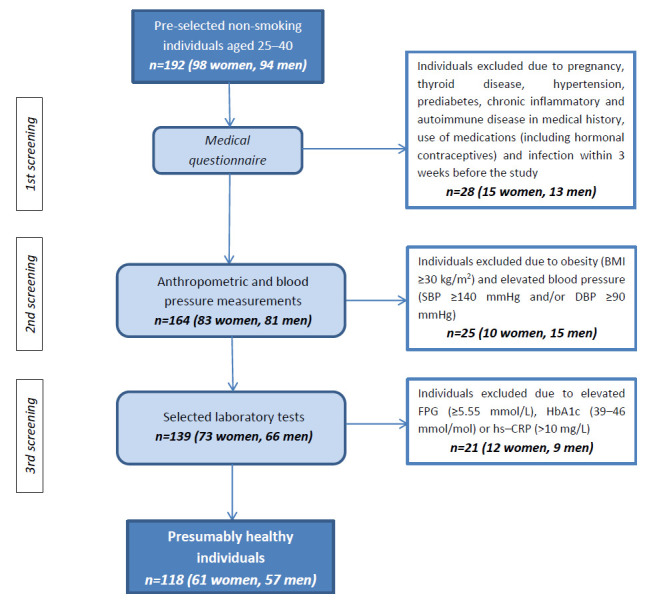
Flow chart of study group selection. Abbreviations: BMI, body mass index; SBP, systolic blood pressure; DBP, diastolic blood pressure; FPG, fasting plasma glucose; HbA1c, glycated hemoglobin; hs-CRP, high sensitivity C-reactive protein.

The cross-sectional descriptive preliminary study included 118 presumably healthy Caucasian individuals (women *n* = 61, 51.7%; men *n* = 57) aged 25–40 years, selected from a general population participating in the diabetes preventive screening program conducted at the Department of Laboratory Diagnostics, Nicolaus Copernicus University, Collegium Medicum in Bydgoszcz, Poland and University Hospital No. 1 in Bydgoszcz, Poland in 2014–2015. From an initial sample of 192 non-smoking individuals aged 25–40 years, 118 were selected based on specific inclusion and exclusion criteria (Figure 1). In the first stage of selection, data from a standard medical questionnaire completed by all participants, regarding current health status and medical history, was analyzed. Then, anthropometric and blood pressure measurements, and selected laboratory tests were performed, based on which the final decision on inclusion or exclusion from the study group was made.

Individuals meeting the following criteria were excluded from the study: current smoker (or quitter in the last 2 years), use of any medications (including hormonal contraception), pregnancy, presence of chronic diseases in medical history: diabetes, CVD, renal disease, thyroid disease, liver disease, inflammatory and autoimmune diseases, and acute infection in the last 3 weeks before the study, arterial hypertension (systolic blood pressure, SBP ≥ 140 mmHg and/or diastolic blood pressure, DBP ≥ 90 mmHg), obesity (defined by body mass index, BMI ≥ 30 kg/m^2^), prediabetes (fasting plasma glucose, FPG ≥ 5.55 mmol/L; ≥100 mg/dL and/or glycated hemoglobin, HbA1c 39–46 mmol/mol; 5.7–6.4%), high sensitivity C-reactive protein (hs-CRP) > 10 mg/L.

Before taking part in the study, all participants were informed about the purpose and method of its conduct and the possibility of withdrawing at any stage, without giving a reason. All subjects provided informed and written consent to participate in the study. The study was approved by the Bioethics Committee of Nicolaus Copernicus University in Torun, Collegium Medicum in Bydgoszcz, Poland (no. KB/627/2010, annexed 23 April 2013) and conducted in accordance with the Declaration of Helsinki.

### 2.2. Anthropometric and Blood Pressure Measurements

Basic anthropometric measurements and blood pressure measurements were performed by trained staff. BMI was calculated as the ratio of body weight (kg) to height (m) squared. To determine waist-hip ratio (WHR), waist circumference (WC) was measured at the midpoint between the lower ribs and the iliac crest, and hip circumference was measured at the widest point around the buttocks. BMI values between 18.5 and 24.9 kg/m^2^ were considered normal weight. Desirable WC was defined as <80 cm for women and <94 cm for men, based on cut-offs for the European population: ≥ [15].

Blood pressure measurements were performed using the M6 Comfort blood pressure monitor (Omron Healthcare, Kyoto, Japan), after 15 min of rest in a sitting position. From each participant, three measurements were taken at 2–3 min rest intervals on the dominant hand and the results were averaged. SBP < 140 mmHg and DBP < 90 mmHg were considered desirable values [16].

### 2.3. Blood Samples Collection and Laboratory Tests

Venous blood samples were drawn in the morning, in a fasting state, 10–12 h after the last meal. Blood was collected successively into three types of the Vacutainer system (Becton Dickinson, Franklin Lakes, NJ, USA) tubes: with clot activator (for serum), EDTA (for HbA1c testing) and sodium fluoride (for FPG measurements). Immediately after collection, tubes for FPG testing were centrifuged at 4 °C for 15 min at 3000 rpm. Serum samples were obtained by centrifuging the tubes after they were completely clotted for 15 min at 3000 rpm.

The following laboratory tests were performed in freshly prepared samples from all participants: FPG, HbA1c, lipid profile, insulin and hs-CRP. The ARCHITECT ci8200 platform (Abbott Laboratories, Chicago, IL, USA) was used for biochemical testing. Calprotectin and adiponectin concentrations were measured in the remaining serum samples, stored in small quantities (300–400 µL) at −80 degrees. The concentration of serum calprotectin was assayed using Human Calprotectin kit (ref. RHK379-02R, BioLab Assays; Brno, Czech Republic), based on the sandwich enzyme-linked immunosorbent assay (ELISA), with detectable range of 1.6 to 100 ng/mL. For serum adiponectin assay Human Adiponectin competitive ELISA kit (ref. RD195023100, BioVendor R&D; Brno, Czech Republic) with a detectable range of 0.1–10.0 µg/mL was used. Serum samples were diluted with an appropriate diluent before testing, according to the procedure specified in the manufacturers’ manuals.

To calculate specific laboratory indicators, the following equations were used: (1) Homeostatic Model Assessment for Insulin Resistance (HOMA-IR) = [glucose (mmol/L) × insulin (µU/mL)]/22.5; (2) non-high-density lipoprotein cholesterol (non-HDL-C) = high-density lipoprotein cholesterol (HDL-C)—total cholesterol (TC); (3) the Sampson formula [17] was applied for low-density lipoprotein cholesterol (LDL-C).

For individual laboratory tests, the following reference/desirable ranges were adopted: FPG 3.89–5.50 mmol/L (70–99 mg/dL) and HbA1c < 39 mmol/mol (<5.7%) [18]; insulin 2–25 µU/mL [19]; HOMA-IR < 2 [20]; hs-CRP < 5 mg/L [21]; TC < 4.9 mmol/L (<190 mg/dL); LDL-C (<115 mg/dL); HDL-C > 1.16 mmol/L (>45 mg/dL) for women and >1.03 mmol/L (>40 mg/dL) for men; TG < 1.7 mmol/L (<150 mg/dL); non-HDL-C < 3.75 mmol/L (<145 mg/dL) [22]. Quartile distribution was calculated for calprotectin: Q1 (25th percentile) 451.4 ng/mL; Q2 (50th percentile) 540.8 ng/mL; Q3 (75th percentile) 634.0 ng/mL; and for adiponectin: Q1 (25th percentile) 6.45 µg/mL; Q2 (50th percentile) 8.11 µg/mL; Q3 (75th percentile) 9.71 µg/mL. Due to the lack of uniform reference values for these parameters, for the purposes of statistical analysis values below Q2 (50th percentile, median) for calprotectin and above Q2 for adiponectin were considered desirable.

### 2.4. Definitions of Cardiometabolic Risk Factors

For the purposes of statistical analysis, cardiometabolic risk factors were adopted based on established cut-off points and clinical recommendations for metabolic syndrome and cardiovascular risk stratification. BMI values between 25.0 and 29.9 kg/m^2^ were considered overweight and abdominal obesity was defined as WC ≥ 80 cm in females and ≥94 cm in males (cut-offs for the European population) [15]. Concentration of hs-CRP ≥ 1 mg/L was considered as moderate/high CVD risk [23]. HOMA-IR > 2 was used as an indicator of early insulin resistance [20]. To reflect atherogenic dyslipidemia, decreased HDL-C < 1.16 mmol/L (<45 mg/dL) for women and <1.03 mmol/L (<40 mg/dL) for men, and increased TG ≥ 1.69 mmol/L (≥150 mg/dL) were used [22]. Adiponectin concentration < 8.11 µg/mL (<median) was considered as decreased. Due to the facts that all participants had normal FPG and normal HbA1c levels, HbA1c > 32 mmol/mol (>5.1%; >median) was applied as an indicator of relatively higher mean glycemia in the study group.

### 2.5. Statistical Analysis

Results were presented as median (Me) and 25th–75th percentile range (1st–3rd quartile, Q1–Q3), due to the non-Gaussian distribution of variables. The Mann–Whitney U test was used to compare the results in two groups. Correlations between serum calprotectin and selected variables were assessed using the Spearman rank test. To compare differences between two percentage values or correlation coefficients, the chi-square (Fisher exact) test was used. To evaluate the relationship between serum calprotectin concentrations and selected cardiometabolic risk factors, logistic regression analysis was performed, with odds ratios calculated per unit increase.

Sample size estimation was based on the literature data reporting correlation coefficients between calprotectin and BMI in the range of r = 0.25–0.52 in metabolic and general population studies [10,24]. Therefore, a minimum of 34–123 subjects were required to achieve 80% power at a two-sided α = 0.05 to detect a moderate correlation between BMI and serum calprotectin.

In all statistical analyses, the level of significance (*p*-value) was set at <0.05. Statistical tests were conducted using Statistica 13.3 software (StatSoft Inc., Tulsa, OK, USA).

## 3. Results

### 3.1. Clinical and Biochemical Characteristics of the Study Participants

General characteristics of the study participants are presented in Table 1 and Table 2. In our group, men had significantly higher values of anthropometric indicators and blood pressure, and a greater tendency to be overweight, when compared to women. Taking into account laboratory tests, FPG, HbA1c, insulin and HOMA-IR, triglycerides (TG) and non-HDL-C were significantly higher in males, whereas HDL-C and adiponectin were lower. Serum calprotectin concentration in study group ranged from 136.8 to 3410.4 ng/mL and was significantly lower in women than in men (*p* = 0.016).

**Figure 2:**
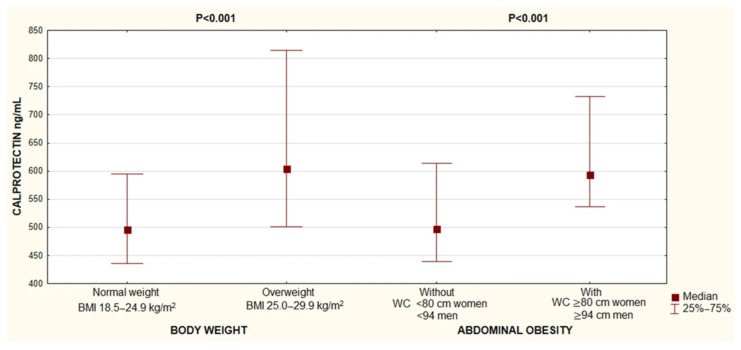
Differences in serum calprotectin concentration, depending on the presence of overweight and abdominal obesity. Abbreviations: BMI, body mass index; WC, waist circumference. *p* values were calculated using the Mann–Whitney U test.

When stratified by BMI, a significantly worse metabolic profile was observed in overweight individuals, related to higher values of FPG, insulinemia and HOMA-IR, hs-CRP, TG, LDL-C and non-HDL-C, as well as lower HDL-C and adiponectin concentration. Significantly higher calprotectin levels were found in overweight subjects (Figure 2), compared to individuals with a normal BMI (median 496.0 vs. 604.3 ng/mL; *p* < 0.001). Similarly, in participants with abdominal obesity, serum calprotectin concentration was significantly increased (median 497.4 vs. 594.0 ng/mL; *p* < 0.001).

### 3.2. Correlation Analysis

Serum calprotectin showed significant positive correlations with anthropometric indicators, particularly BMI, CRP, TG and non-HDL-C, as well as laboratory markers of glucose metabolism: HbA1c, insulin and HOMA-IR in the whole group (Table 3). Negative correlations were observed for HDL-C and adiponectin. In women, strongest correlations were noted for CRP, BMI, WC and WHR, while in men for CRP, BMI, TG and HbA1c, respectively. However, the correlation coefficients did not differ significantly between sexes. After taking into account the division by BMI, in the group with normal weight significant correlations were found for BMI (R = 0.34; *p* = 0.002), WC (R = 0.29; *p* = 0.012), and HDL-C (R = −0.27; *p* = 0.019), while in overweight subjects for hs-CRP (R = 0.44; *p* = 0.002), insulin (R = 0.38; *p* = 0.007), HOMA-IR (R = 0.36; *p* = 0.011), HbA1c (R = 0.30; *p* = 0.024) and TG (R = 0.29; *p* = 0.043).

### 3.3. Relationship Between Serum Calprotectin Concentration and Risk Factors for Metabolic Disorders

Selected variables were evaluated as predictors of relatively high concentration of serum calprotectin, equal to or above the median value (≥540.8 ng/mL; Table 4). In univariable regression analysis, higher levels of calprotectin were positively associated with the occurrence of cardiometabolic risk factors: overweight and abdominal obesity, CRP concentration ≥ 1 mg/L (indicating low-grade inflammation), increased HOMA-IR (related to insulin resistance), higher values of HbA1c (>32 mmol/mol; >median), decreased HDL-C and hypertriglyceridemia (≥1.69 mmol/L; ≥150 mg/dL). After adjustment for sex and BMI, increased values of CRP, HbA1c, HOMA-IR and TG remained significant predictors for high calprotectin concentration. However, in the model adjusted additionally for CRP, significant association was observed only for HbA1c and HOMA-IR.

**Figure 3:**
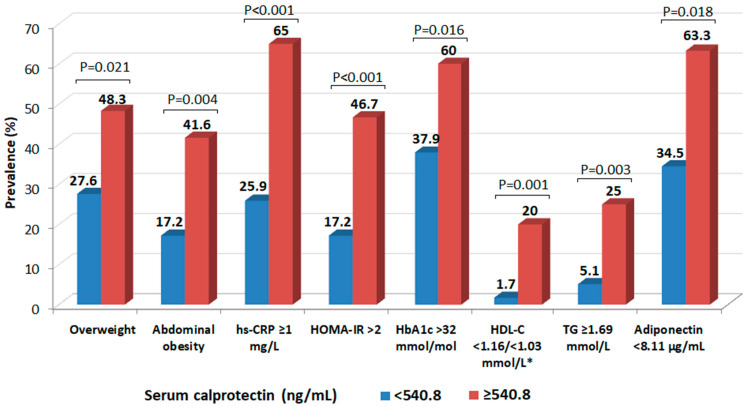
The incidence of cardiovascular and metabolic risk factors, depending on the calprotectin concentration. Abbreviations: hs-CRP, high sensitivity C-reactive protein; HOMA-IR, Homeostatic Model Assessment for Insulin Resistance; HbA1c, glycated hemoglobin; HDL-C, high-density lipoprotein cholesterol; TG, triglycerides. * Women/Men. *p* values were calculated using the chi-square (Fisher exact) test.

In further analysis, we assessed the prevalence of selected cardiometabolic risk factors, depending on serum calprotectin concentration (Figure 3). Subjects with higher calprotectin concentration (≥540.8 ng/mL; ≥median) were significantly more likely to be overweight. The incidence of increased waist circumference, indicating abdominal obesity, and CRP ≥ 1 mg/L, were approximately 2.5 times higher than in subjects with lower calprotectin levels (<540.8 ng/mL). Lower adiponectin concentration (<8.11 µg/mL; <median), HbA1c > 32 mmol/mol (determining higher average glycemia) and HOMA-IR > 2 were significantly more frequent in subjects with higher calprotectin levels. Considering atherogenic dyslipidemia, the prevalence of subjects with decreased HDL-C (<1.16 mmol/L in women and <1.03 mmol/L in men) and elevated TG (≥1.69 mmol/L) was relevantly several times lower in participants with lower calprotectin concentrations.

## 4. Discussion

Our preliminary results demonstrated that in presumably healthy young adults, serum calprotectin is significantly increased in subjects with overweight and abdominal obesity, compared with lean ones. Concentration of serum calprotectin also showed a positive association with CRP, HbA1c, early insulin resistance, unfavorable changes in lipid profile and low adiponectin levels, suggesting its potential role as a laboratory biomarker of early disturbances in glucose and lipid metabolism. In the available publications, the most extensive discussion has been given to the relationship between serum calprotectin and the occurrence of obesity and the risk of type 2 diabetes, while few studies refer to a group of healthy people without noticeable cardiometabolic disorders. In the previously mentioned study by Catalan et al. [10], significantly increased serum calprotectin levels were found in both obese normoglycemic and T2DM subjects, when compared to lean individuals. Interestingly, the authors observed a significant decrease in calprotectin levels in obese patients after weight loss achieved by Roux-en-Y gastric bypass (RYGB) and the differences were positively correlated with the differences in the WHR, as well as in the concentrations of the inflammatory markers, including CRP. In the PREVEND, a prospective population-based cohort study, serum calprotectin was associated with new-onset T2DM in the general population, with the highest rate in subjects with calprotectin concentration in the 3rd tertile (>0.60 mg/L) [25]. In a Danish study in a group of 305 T2DM patients without known CVD, serum calprotectin concentration was significantly higher compared to healthy controls (3754 vs. 2437 ng/mL; *p* <  0.0001), and moreover was also significantly higher in patients with metabolic syndrome [24]. In the linear regression analysis serum calprotectin was independently associated with BMI, C-reactive protein, and HDL-C, which partially supports the results from our preliminary study. Calprotectin concentration in serum can also be elevated in subjects with impaired glucose tolerance and positively associated with anthropometric indices, as well as with insulin resistance, independently of BMI and age [26]. Interestingly, the authors observed similar findings for calprotectin determined in urine. It was also observed that weight loss led to decrease in circulating calprotectin, in parallel to fasting glucose and HOMA-IR values. Studies conducted in children also indicate a strong relationship between calprotectin concentration and BMI as independent predictor [27] and moreover with impaired fasting glucose and insulin resistance [28]. Although the cited studies did not directly examine calprotectin activity in adipose tissue, it can be hypothesized that excessive visceral adipose tissue itself increases calprotectin levels by stimulating inflammation and increasing chemotaxis of inflammatory cells, which are the main source of calprotectin. Subsequently, excessive proinflammatory activation may lead to dysregulation of adipocytokine secretion, with a predominance of those with proinflammatory properties, which further contributes to the progression of systemic inflammation, promoting insulin resistance, atherosclerosis, and the development of CVD [9]. In our results the logistic regression analysis did not show that low adiponectin concentration was an independent predictor of increased calprotectin concentration. However, it is worth emphasizing the significant negative correlation between calprotectin and adiponectin and the almost twice as frequent occurrence of low adiponectin concentrations in patients with high calprotectin levels. Despite the previously cited study [10] which indicated a negative, but not statistically significant, correlation between serum calprotectin and adiponectin (R= −0.27; *p* = 0.252), there are no detailed studies on the relationship between serum calprotectin and adiponectin in the context of obesity or metabolic syndrome. However, it can be assumed that the concentration of adiponectin, as an adipocytokine with proven anti-inflammatory and insulin sensitivity increasing effects [1,2], might be inversely associated with calprotectin, related to low-grade inflammation and affecting glycemia.

In our study group, we did not directly assess body composition and adiposity, including visceral adipose tissue content and their relationship with serum calprotectin concentration. The study was limited to anthropometric indicators, which indirectly reflect the tendency for adipose tissue accumulation in the abdominal area. Numerous studies confirm the existence of relationships between the level of visceral and subcutaneous adipose tissue, various inflammatory markers and increased cardiometabolic risk [29,30,31]. However, data on serum calprotectin are quite limited in this regard. In the study by Sekimoto et al. [11] serum calprotectin showed relevant positive correlation with basic anthropometric measurements (BMI, WC) and with visceral (VFA) and subcutaneous fat area (SFA), measured by computed tomography method, in Japanese men with abdominal obesity. Multiple regression analysis identified VFA and SFA as independent determinants of calprotectin concentration. The previously described calprotectin expression in VAT [10] was particularly intense in patients with obesity and T2DM, compared to lean and normoglycemic individuals, and the circulating calprotectin concentration correlated significantly with the expression and concentration of other proinflammatory adipocytokines and cytokines synthesized in adipocytes (e.g., leptin, IL-6, TNF-alpha). Recent studies emphasize the importance of epicardial adipose tissue (EAT) measurements as a component of VAT, which shows significant association with obesity, insulin resistance and T2DM, linking the endocrine activity of adipocytes with inflammation and cardiovascular complications [32,33,34]. In this context, extending research to assess the relationship between serum calprotectin and EAT seems to be valuable for understanding the mechanisms that increase cardiometabolic risk.

In this preliminary report, we noted a slight, but statistically significant association of serum calprotectin with triglycerides and HDL-C, while the relationship with HbA1c and HOMA-IR seems to be stronger. This may suggest that in our cohort, higher serum calprotectin levels may better reflect early disturbances related to glycemic control, than to dyslipidemia. Studies do not indicate a direct relationship between serum calprotectin, lipid profile and risk of atherosclerosis; however, in a study by Ionita et al. [35] higher levels of calprotectin subunit (described as MRP-14) were found in atherosclerotic lesions with high macrophage staining, large lipid core and high levels of IL-6 and interleukin 8 (IL-8). Higher serum calprotectin levels were also observed in a study on diabetic subjects with coronary artery disease (CAD), compared to those without CAD and these also correlated with disease severity [36]. What is worth emphasizing, in a group without CAD, serum calprotectin was associated with carotid intima media thickness (IMT) in both diabetic and non-diabetic individuals. These results potentially indicate that calprotectin is involved in atherosclerotic plaques formation and progression, and together with lipid and/or glycemic abnormalities induced by inflammation in adipose tissue, may accelerate the development of cardiovascular disease. Observations from the Dallas Heart Study [12] revealed that high serum calprotectin was related to higher HbA1c, very low-density lipoprotein cholesterol, triglycerides, lower high-density lipoprotein cholesterol and cholesterol efflux capacity, and with almost 2-fold increased risk of atherosclerotic cardiovascular disease (ASCVD) events over 8 years (hazard ratio: 1.98; 95% CI: 1.54–2.53). In the in vitro model, the authors indicated that elevated calprotectin concentration impairs coronary endothelial integrity and reduces nitric oxide production, potentially leading to progression ASCVD progression.

In our study we observed a relevant difference in serum calprotectin concentration between women and men. In general, women in our study were characterized by a lower incidence of overweight and increased waist circumference, as well as a better metabolic profile and lower hs-CRP in laboratory tests, which may indirectly indicate less severe inflammation resulting from lower adiposity. There is no clear evidence in the literature of significant differences in serum calprotectin concentration between the sexes. Zuo et al. observed an association between elevated calprotectin and male sex [12], while a study in children indicated higher levels in females [28] and a study in elderly subjects showed no relevant differences [37]. At this point, attention should be paid to the potential changes in calprotectin concentration in women during the menstrual cycle, which may distort the assessment of cardiometabolic risk. Even though there is no research available on this issue, it can be assumed that calprotectin, as a marker of inflammation, may be elevated, similarly to CRP, during menstruation and the early follicular phase of the cycle [38], and in women using hormone contraceptives [39]. However, our study included only females who did not use any hormonal birth control and they were instructed to perform blood tests at least 3–4 days after menstruation, therefore the impact of hormonal changes may be considered insignificant.

To our knowledge, this preliminary study might be one of the few publications evaluating the association of serum calprotectin with selected cardiovascular and metabolic risk factors in normoglycemic and non-obese young adults, highlighting the potential diagnostic significance as a biomarker of inflammation and initial metabolic impairment. However, our study has several important limitations: (1) the study group is small; (2) the participants were homogeneous in age and ethnicity, which may not reflect relationships that would occur in a more diverse population; (3) the cross-sectional design of this study does not allow for an assessment of a causal relationship between serum calprotectin and metabolic abnormalities; (4) comparison of results from different studies may be difficult due to the differences in assay methods for determining serum calprotectin concentration and their analytical performance. Future perspectives for the study include extending the study to a larger, more representative population and a follow-up study, which would allow for a proper assessment of the relationship between baseline calprotectin concentration and the risk of cardiometabolic diseases in the future. It also seems important to include different age groups and body composition measurements, especially adipose tissue content and physical activity levels, in future studies. This strategy would allow for a more accurate assessment of the relationship between body fat distribution, serum calprotectin concentration, and other biomarkers and the risk of diabetes and cardiovascular disease in the general population.

## 5. Conclusions

Our preliminary study reported a significant relationship between serum calprotectin concentration and selected cardiometabolic risk factors: overweight, laboratory markers of glycemia, insulin resistance, inflammation, and dyslipidemia, in young, presumably healthy, non-obese adults. This emphasizes the potential significance of serum calprotectin measurements as a laboratory biomarker of initial metabolic impairment. However, due to important limitations of the study, our findings require verification in a large population study.

## Figures and Tables

**Table 1:** Comparison of clinical variables by sex and BMI values.

Variables	All (n = 118)	Women (n = 61)	Men (n = 57)	*p* *	Normal Weight (n = 90)	Overweight (n = 28)	*p* **
Age (years)	30.5 (26–34)	31 (26–36)	30 (27–33)	0.901	29 (26–33)	33 (28–36)	0.001
BMI (kg/m^2^)	23.8 (21.1–25.9)	21.6 (20.3–23.8)	24.5 (22.9–26.7)	<0.001	21.3 (20.1–23.4)	26.8 (25.7–28.1)	<0.001
Overweight (n; [%])	28; [23.7]	10; [16.4]	18; [31.6]	0.052	---	---	---
WC (cm)	82.5 (72–92)	72 (69–79)	91 (87–96)	<0.001	73 (69–82)	92 (89–97)	<0.001
WHR	0.83 (0.76–0.88)	0.77 (0.74–0.79)	0.88 (0.86–0.91)	<0.001	0.77 (0.74–0.84)	0.88 (0.84–0.92)	<0.001
Abdominal obesity (n; [%])	32; [27.1]	13; [21.3]	19; [33.3]	0.143	12; [13.3]	20 [71.4]	<0.001
SBP (mmHg)	116 (104–128)	110 (102–119)	126 (115–135)	<0.001	118 (111–128)	128 (123–135)	<0.001
DBP (mmHg)	77 (68–84)	75 (66–82)	82 (73–88)	<0.001	79 (75–85)	82 (78–87)	0.017

Results are presented as median and Q1–Q3 ranges (25th–75th percentile). Abbreviations: BMI, body mass index; WC, waist circumference; WHR, waist–hip ratio; SBP, systolic blood pressure; DBP, diastolic blood pressure. P, the statistical significance of differences between groups was calculated using the Mann–Whitney U test; * women vs. men, ** normal weight vs. overweight.

**Table 2:** Comparison of laboratory tests results by sex and BMI values.

Variables	All (n = 118)	Women (n = 61)	Men (n = 57)	*p* *	Normal Weight (n = 90)	Overweight (n = 28)	*p* **
FPG (mmol/L)	5.00 (4.83–5.33)	4.94 (4.72–5.16)	5.22 (5.00–5.44)	<0.001	5.06 (4.78–5.28)	5.17 (4.89–5.39)	0.001
HbA1c (mmol/mol)	32 (30–34)	32 (29–33)	33 (31–36)	<0.001	32 (30–34)	33 (30–36)	0.423
Insulin (µU/mL)	7.30 (5.37–10.01)	6.44 (5.03–8.14)	8.65 (6.27–11.21)	0.001	6.82 (4.68–8.77)	7.89 (5.95–11.06)	0.011
HOMA-IR	1.67 (1.20–2.25)	1.46 (1.13–1.79)	1.90 (1.41–2.54)	<0.001	1.48 (1.11–1.94)	1.86 (1.35–2.50)	0.008
hs-CRP (mg/L)	0.65 (0.40–1.50)	0.50 (0.30–1.80)	0.75 (0.40–1.40)	0.374	0.50 (0.30–1.40)	1.00 (0.55–1.70)	0.007
TC (mmol/L)	4.91 (4.40–5.40)	4.96 (4.40–5.30)	4.89 (4.40–5.40)	0.821	4.86 (4.14–5.20)	4.99 (4.42–5.71)	0.043
LDL-C (mmol/L)	2.97 (2.59–3.49)	2.87 (2.40–3.21)	3.13 (2.61–3.67)	0.062	2.87 (2.35–3.21)	3.23 (2.66–3.83)	0.004
HDL-C (mmol/L)	1.40 (1.16–1.60)	1.55 (1.34–1.71)	1.21 (1.11–1.42)	<0.001	1.47 (1.34–1.68)	1.19 (1.09–1.42)	<0.001
non-HDL-C (mmol/L)	3.46 (3.00–4.03)	3.36 (2.84–3.77)	3.75 (3.23–4.16)	0.010	3.36 (2.71–3.77)	3.88 (3.26–4.39)	<0.001
TG (mmol/L)	0.92 (0.70–1.31)	0.81 (0.63–1.08)	1.02 (0.83–1.77)	<0.001	0.87 (0.70–1.12)	1.41 (0.98–1.79)	<0.001
Calprotectin (ng/mL)	540.8 (451.4–634.0)	528.6 (436.2–608.8)	593.6 (483.6–689.0)	0.016	496.0 (436.2–595.2)	604.3 (500.6–814.5)	<0.001
Adiponectin (µg/mL)	8.11 (6.45–9.71)	8.99 (7.48–10.45)	7.01 (5.36–8.43)	<0.001	8.77 (7.15–10.37)	6.83 (5.63–8.46)	<0.001

Results are presented as median and Q1–Q3 ranges (25th–75th percentile). Abbreviations: FPG, fasting plasma glucose; HbA1c, glycated hemoglobin; HOMA-IR, Homeostatic Model Assessment for Insulin Resistance; hs-CRP, high sensitivity C-reactive protein; TC, total cholesterol; LDL-C, low-density lipoprotein cholesterol HDL-C, high-density lipoprotein cholesterol; non-HDL-C, non-high-density lipoprotein cholesterol; TG, triglycerides. P, the statistical significance of differences between groups was calculated using the Mann–Whitney U test; * women vs. men, ** normal weight vs. overweight.

**Table 3:** Correlation of serum calprotectin with measured variables in the study group (Spearman’s R correlation coefficients).

Variables	All (n = 118)		Women (n = 61)		Men (n = 57)		*p* *
	R	*p*	R	*p*	R	*p*	Women vs. Men
Age	0.06	0.535	0.13	0.310	0.02	0.892	0.559
BMI	0.44	<0.001	0.48	<0.001	0.37	0.004	0.478
WC	0.38	<0.001	0.46	<0.001	0.18	0.183	0.098
WHR	0.32	<0.001	0.38	<0.001	0.12	0.356	0.142
SBP	0.21	0.021	0.03	0.824	0.22	0.086	0.308
DBP	0.06	0.538	0.03	0.828	0.08	0.557	0.791
FPG	0.15	0.094	0.18	0.161	0.01	0.109	0.365
HbA1c	0.29	0.001	0.14	0.271	0.33	0.010	0.288
Insulin	0.29	0.001	0.28	0.023	0.20	0.130	0.654
HOMA-IR	0.30	<0.001	0.30	0.017	0.15	0.253	0.404
hs-CRP	0.48	<0.001	0.58	<0.001	0.39	0.002	0.188
TC	0.11	0.289	0.08	0.537	0.07	0.615	0.958
LDL-C	0.05	0.603	0.06	0.609	0.09	0.517	0.873
HDL-C	−0.29	<0.001	−0.13	0.293	−0.32	0.012	0.290
Non-HDL-C	0.21	0.017	0.21	0.099	0.17	0.195	0.827
TG	0.34	<0.001	0.24	0.061	0.36	0.004	0.486
Adiponectin	−0.24	0.008	−0.13	0.294	−0.20	0.135	0.704

Abbreviations: BMI, body mass index; WC, waist circumference; WHR, waist–hip ratio; SBP, systolic blood pressure; DBP, diastolic blood pressure; FPG, fasting plasma glucose; HbA1c, glycated hemoglobin; HOMA-IR, Homeostatic Model Assessment for Insulin Resistance; hs-CRP, high sensitivity C-reactive protein; TC, total cholesterol; LDL-C, low-density lipoprotein cholesterol HDL-C, high-density lipoprotein cholesterol; non-HDL-C, non-high-density lipoprotein cholesterol; TG, triglycerides. * The statistical significance of differences calculated using the chi-square (Fisher exact) test.

**Table 4:** Selected predictors of high (≥540.8 ng/mL; ≥median) serum calprotectin concentration in logistic regression analysis.

Risk Factors	Unadjusted		Adjusted for Sex and BMI		Adjusted for Sex, BMI and CRP	
	OR (95% CI)	*p*	OR (95% CI)	*p*	OR (95% CI)	*p*
Overweight (BMI > 25 kg/m^2^)	2.529 (1.194–5.357)	0.015	---	---	1.787 (0.732–4.364) **	0.203
Abdominal obesity (WC ≥ 80/≥94 cm, F/M)	3.217 (1.409–7.348)	0.006	1.518 (0.549–4.192)	0.421	1.274 (0.452–3.590)	0.647
hs-CRP ≥ 1 mg/L	5.000 (2.307–10.835)	<0.001	4.788 (2.111–10.864)	<0.001	---	---
HbA1c > 32 mmol/mol *	2.166 (1.018–4.608)	0.021	2.484 (1.089–5.668)	0.013	3.000 (1.256–7.169)	0.011
HOMA-IR > 2	4.394 (1.892–10.204)	<0.001	3.641 (1.465–9.052)	0.005	3.233 (1.273–8.211)	0.014
TG ≥ 1.69 mmol/L	1.609 (1.184–2.401)	0.010	1.323 (1.017–1.583)	0.047	1.255 (0.801–1.323)	0.099
HDL-C < 1.16/<1.03 mmol/L (F/M)	1.127 (1.079–1.412)	0.031	1.310 (0.934–1.542)	0.114	1.064 (0.853–1.862)	0.314
Adiponectin < 8.11 µg/mL *	1.647 (0.803–3.377)	0.174	1.213 (0.526–2.799)	0.650	0.975 (0.409–2.324)	0.955

Abbreviations: BMI, body mass index; WC, waist circumference; F, female; M, male; hs-CRP, high sensitivity C-reactive protein; HbA1c, glycated hemoglobin; HOMA-IR, Homeostatic Model Assessment for Insulin Resistance; TG, triglycerides; HDL-C, high-density lipoprotein cholesterol. * Median values. ** Adjusted for sex and CRP.

## Data Availability

The data can be made available upon reasonable request—please contact the correspondence author. The data are not publicly available due to fact that it contains information that could compromise the privacy of research participants.

## Abbreviations

The following abbreviations are used in this manuscript:

T2DM	type 2 diabetes
CVD	cardiovascular disease
CRP	C-reactive protein
IL-6	interleukin 6
IL-1	interleukin 1
IBD	inflammatory bowel disease
VAT	visceral adipose tissue
BMI	body mass index
FPG	fasting plasma glucose
HbA1c	glycated hemoglobin
SBP	systolic blood pressure
DBP	diastolic blood pressure
WC	waist circumference
WHR	waist-hip ratio
hs-CRP	high sensitivity C-reactive protein
ELISA	enzyme-linked immunosorbent assay
HOMA-IR	Homeostatic Model Assessment for Insulin Resistance
LDL-C	low-density lipoprotein cholesterol
non-HDL-C	non-high-density lipoprotein cholesterol
HDL-C	high-density lipoprotein cholesterol
TC	total cholesterol
TG	triglycerides
RYGB	Roux-en-Y gastric bypass
IL-8	interleukin 8
VFA	visceral fat area
SFA	subcutaneous fat area
EAT	epicardial adipose tissue
CAD	coronary artery disease
IMT	intima media thickness
ASCVD	atherosclerotic cardiovascular disease

## References

[1] Valenzuela P.L., Carrera-Bastos P., Castillo-García A., Lieberman D.E., Santos-Lozano A., Lucia A. (2023). Obesity and the risk of cardiometabolic diseases. Nat. Rev. Cardiol..

[2] Savulescu-Fiedler I., Mihalcea R., Dragosloveanu S., Scheau C., Baz R.O., Caruntu A., Scheau A.-E., Caruntu C., Benea S.N. (2024). The Interplay between Obesity and Inflammation. Life.

[3] Wu H., Ballantyne C.M. (2020). Metabolic Inflammation and Insulin Resistance in Obesity. Circ. Res..

[4] Ferreira J.P., Vasques-Nóvoa F., Neves J.S., Zannad F., Leite-Moreira A. (2024). Comparison of interleukin-6 and high-sensitivity C-reactive protein for cardiovascular risk assessment: Findings from the MESA study. Atherosclerosis.

[5] Jukic A., Bakiri L., Wagner E.F., Tilg H., Adolph T.E. (2021). Calprotectin: From biomarker to biological function. Gut.

[6] Khaki-Khatibi F., Qujeq D., Kashifard M., Moein S., Maniati M., Vaghari-Tabari M. (2020). Calprotectin in inflammatory bowel disease. Clin. Chim. Acta.

[7] Dajti E., Frazzoni L., Iascone V., Secco M., Vestito A., Fuccio L., Eusebi L.H., Fusaroli P., Rizzello F., Calabrese C. (2023). Systematic review with meta-analysis: Diagnostic performance of faecal calprotectin in distinguishing inflammatory bowel disease from irritable bowel syndrome in adults. Aliment. Pharmacol. Ther..

[8] Shi J.-T., Chen N., Xu J., Goyal H., Wu Z.-Q., Zhang J.-X., Xu H.-G. (2023). Diagnostic Accuracy of Fecal Calprotectin for Predicting Relapse in Inflammatory Bowel Disease: A Meta-Analysis. J. Clin. Med..

[9] Manfredi M., Van Hoovels L., Benucci M., De Luca R., Coccia C., Bernardini P., Russo E., Amedei A., Guiducci S., Grossi V. (2023). Circulating Calprotectin (cCLP) in autoimmune diseases. Autoimmun. Rev..

[10] Wirtz T.H., Buendgens L., Weiskirchen R., Loosen S.H., Haehnsen N., Puengel T., Abu Jhaisha S., Brozat J.F., Hohlstein P., Koek G. (2020). Association of Serum Calprotectin Concentrations with Mortality in Critically Ill and Septic Patients. Diagnostics.

[11] Kruzliak P., Novák J., Novák M., Fodor G.J. (2014). Role of calprotectin in cardiometabolic diseases. Cytokine Growth Factor Rev..

[12] Catalán V., Gómez-Ambrosi J., Rodríguez A., Ramírez B., Rotellar F., Valentí V., Silva C., Gil M.J., Fernández-Real J.M., Salvador J. (2011). Increased levels of calprotectin in obesity are related to macrophage content: Impact on inflammation and effect of weight loss. Mol. Med..

[13] Sekimoto R., Kishida K., Nakatsuji H., Nakagawa T., Funahashi T., Shimomura I. (2012). High circulating levels of S100A8/A9 complex (calprotectin) in male Japanese with abdominal adiposity and dysregulated expression of S100A8 and S100A9 in adipose tissues of obese mice. Biochem. Biophys. Res. Commun..

[14] Zuo Y., NaveenKumar S.K., Navaz S., Liang W., Sugur K., Kmetova K., Ayers C.R., Kluge L., Chong E., Shah A.M. (2025). Epidemiological and Translational Study of Calprotectin and Atherosclerotic Cardiovascular Disease. JAMA Cardiol..

[15] Alberti K.G., Eckel R.H., Grundy S.M., Zimmet P.Z., Cleeman J.I., Donato K.A., Fruchart J.C., James W.P., Loria C.M., Smith S.C. (2009). Harmonizing the metabolic syndrome: A joint interim statement of the International Diabetes Federation Task Force on Epidemiology and Prevention; National Heart, Lung, and Blood Institute; American Heart Association; World Heart Federation; International Atherosclerosis Society; and International Association for the Study of Obesity. Circulation.

[16] McEvoy J.W., McCarthy C.P., Bruno R.M., Brouwers S., Canavan M.D., Ceconi C., Christodorescu R.M., Daskalopoulou S.S., Ferro C.J., Gerdts E. (2024). 2024 ESC Guidelines for the management of elevated blood pressure and hypertension. Eur. Heart J..

[17] Sampson M., Ling C., Sun Q., Harb R., Ashmaig M., Warnick R., Sethi A., Fleming J.K., Otvos J.D., Meeusen J.W. (2020). A New Equation for Calculation of Low-Density Lipoprotein Cholesterol in Patients With Normolipidemia and/or Hypertriglyceridemia. JAMA Cardiol..

[18] American Diabetes Association Professional Practice Committee (2025). 2. Diagnosis and Classification of Diabetes: Standards of Care in Diabetes—2025. Diabetes Care.

[19] Abbott Laboratories (2015). Architect Insulin [Package Insert].

[20] Gayoso-Diz P., Otero-González A., Rodriguez-Alvarez M.X., Gude F., García F., De Francisco A., Quintela A.G. (2013). Insulin resistance (HOMA-IR) cut-off values and the metabolic syndrome in a general adult population: Effect of gender and age: EPIRCE cross-sectional study. BMC Endocr. Disord..

[21] Abbott Laboratories (2015). Multigent CRP Vario [Package Insert].

[22] Mach F., Baigent C., Catapano A.L., Koskinas K.C., Casula M., Badimon L., Chapman M.J., De Backer G.G., Delgado V., Ference B.A. (2020). 2019 ESC/EAS Guidelines for the management of dyslipidaemias: Lipid modification to reduce cardiovascular risk. Eur. Heart J..

[23] Biasucci L.M., CDC, AHA (2004). CDC/AHA Workshop on Markers of Inflammation and Cardiovascular Disease: Application to Clinical and Public Health Practice: Clinical use of inflammatory markers in patients with cardiovascular diseases: A background paper. Circulation.

[24] Pedersen L., Nybo M., Poulsen M.K., Henriksen J.E., Dahl J., Rasmussen L.M. (2014). Plasma calprotectin and its association with cardiovascular disease manifestations, obesity and the metabolic syndrome in type 2 diabetes mellitus patients. BMC Cardiovasc. Disord..

[25] Bourgonje A.R., Bourgonje M.F., Sokooti S., la Bastide-van Gemert S., Nilsen T., Hidden C., Gansevoort R.T., Mulder D.J., Hillebrands J.L., Bakker S.J.L. (2024). Plasma Calprotectin and New-onset Type 2 Diabetes in the General Population: A Prospective Cohort Study. J. Clin. Endocrinol. Metab..

[26] Ortega F.J., Sabater M., Moreno-Navarrete J.M., Pueyo N., Botas P., Delgado E., Ricart W., Frühbeck G., Fernández-Real J.M. (2012). Serum and urinary concentrations of calprotectin as markers of insulin resistance and type 2 diabetes. Eur. J. Endocrinol..

[27] Grand A., Rochette E., Dutheil F., Gozal D., Calcaterra V., Berni Canani R., Cobanoglu N., Derikx J.P.M., Terrin G., Pereira B. (2020). Body Mass Index and Calprotectin Blood Level Correlation in Healthy Children: An Individual Patient Data Meta-Analysis. J. Clin. Med..

[28] Calcaterra V., De Amici M., Leonard M.M., De Silvestri A., Pelizzo G., Buttari N., Michev A., Leggio M., Larizza D., Cena H. (2018). Serum Calprotectin Level in Children: Marker of Obesity and its Metabolic Complications. Ann. Nutr. Metab..

[29] Yu J.Y., Choi W.J., Lee H.S., Lee J.W. (2019). Relationship between inflammatory markers and visceral obesity in obese and overweight Korean adults: An observational study. Medicine.

[30] Pereira-Manfro W.F., Lima G.R., Nogueira Neto J.F., Portugal M.R.C., Milagres L.G., Bezerra F.F., Faerstein E., Koury J.C. (2021). Association between visceral/subcutaneous adipose tissue ratio and plasma inflammatory markers and score for cardiovascular risk prediction in a Brazilian cohort: Pró-Saúde Study. Braz. J. Med. Biol. Res..

[31] Chait A., den Hartigh L.J. (2020). Adipose Tissue Distribution, Inflammation and Its Metabolic Consequences, Including Diabetes and Cardiovascular Disease. Front. Cardiovasc. Med..

[32] Ma Z.Y., Duan H., Han D., He B., Xie X.J., Lu L., Jiang J., Li R.H. (2023). Epicardial fat in patients with metabolic syndrome: A systematic review and meta-analysis. Eur. J. Radiol..

[33] Kuleta K., Krauz K., Żmuda J., Momot K., Zarębiński M., Poprawa I., Wojciechowska M. (2025). Pharmacological and Non-Pharmacological Interventions in Diabetes Mellitus: Effects on Epicardial Adipose Tissue. Int. J. Mol. Sci..

[34] Sedaia E.S., Revenco V., Ochisor V. (2022). The role of metabolic syndrome, visceral obesity and insulin resistance in right and left ventricular hypertrophy. Atherosclerosis.

[35] Ionita M.G., Vink A., Dijke I.E., Laman J.D., Peeters W., van der Kraak P.H., Moll F.L., de Vries J.P., Pasterkamp G., de Kleijn D.P. (2009). High levels of myeloid-related protein 14 in human atherosclerotic plaques correlate with the characteristics of rupture-prone lesions. Arterioscler. Thromb. Vasc. Biol..

[36] Peng W.H., Jian W.X., Li H.L., Hou L., Wei Y.D., Li W.M., Xu Y.W. (2011). Increased serum myeloid-related protein 8/14 level is associated with atherosclerosis in type 2 diabetic patients. Cardiovasc. Diabetol..

[37] Nilsen T., Sundström J., Lind L., Larsson A. (2014). Serum calprotectin levels in elderly males and females without bacterial or viral infections. Clin. Biochem..

[38] Gursoy A.Y., Caglar G.S., Kiseli M., Pabuccu E., Candar T., Demirtas S. (2015). CRP at early follicular phase of menstrual cycle can cause misinterpretation for cardiovascular risk assessment. Interv. Med. Appl. Sci..

[39] Masama C., Jarkas D.A., Thaw E., Daneshmend A.Z.B., Franklyn S.I., Beaurepaire C., McQuaid R.J. (2022). Hormone contraceptive use in young women: Altered mood states, neuroendocrine and inflammatory biomarkers. Horm. Behav..

